# Status and influencing factors of farmers’ private investment in the prevention and control of sheep brucellosis in China: A cross-sectional study

**DOI:** 10.1371/journal.pntd.0007285

**Published:** 2019-03-25

**Authors:** Heng Zeng, YouMing Wang, XiangDong Sun, Ping Liu, QuanGang Xu, Duan Huang, Lu Gao, ShiBing You, BaoXu Huang

**Affiliations:** 1 School of Business, Hubei University, Wuhan, Hubei Province, China; 2 China Animal Health and Epidemiology Center, Qingdao, Shandong Province, China; 3 Institute of Geodesy and Geophysics, Chinese Academy of Sciences, Wuhan, Hubei Province, China; 4 School of Economics and Management, Wuhan University, Wuhan, Hubei Province, China; University of Iowa, UNITED STATES

## Abstract

**Background:**

Brucellosis is one of the most common zoonoses worldwide, causing direct losses to the livestock industry and threatening human health. Little is known about the status and factors affecting farmers’ private investment in the prevention and control of sheep brucellosis in China.

**Methodology/Principal findings:**

From April to October 2017, a cross-sectional, house-based study was conducted in 7 Chinese provinces. A total of 1037 households included in the study were analyzed. The average amount of private investment in the prevention and control of brucellosis was $0.73±0.54 per sheep. Multivariable analysis showed that factors facilitating private investment included older age of householder (OR = 1.07, 95%CI: 1.03–1.11), herd size >100 (OR = 2.49, 95%CI: 1.38–4.51), a higher percentage of income from sheep farming comparing to the total household income (OR = 1.14, 95%CI: 1.11–1.16), higher score of brucellosis knowledge (OR = 3.85, 95%CI: 1.40–10.51), actively learning related knowledge (OR = 2.98, 95%CI: 1.55–5.74), actively participating in related training courses (OR = 3.07, 95%CI: 1.52–6.18), care about other people’s attitudes (OR = 1.75, 95%CI: 1.35–2.28), concern about the health of neighbors’ livestock (OR = 1.75, 95%CI: 1.23–2.51). The analysis found a discouraging factor for private investment, supporting culling policy (OR = 0.67, 95%CI: 0.49–0.91).

**Conclusions/Significance:**

In addition to providing interventions related to farmers’ knowledge, attitudes and practices, guidance must be offered to help farmers understanding the importance of private investment in the prevention and control of brucellosis.

## Author summary

Brucellosis is one of the most common zoonoses worldwide. The disease is endemic in China and the prevention and control of brucellosis mainly depends on government investment. From the perspective of economic theory, private investment can help supplement public investment. Thus, it is important to describe the status of farmers’ private investment in brucellosis prevention and control in epidemic areas and to explore the factors influencing this investment.

## Introduction

Brucellosis is a bacterial disease caused by *Brucella*. Sheep, goats, cattle, pigs, dogs and wild animals are susceptible to the disease [1]. Brucellosis impacts livestock productivity and contaminates milk sources [2]. One study [3] revealed that a loss of approximately 3.4 billion dollars was caused by brucellosis in 2015 in animal husbandry in India. Brucellosis is one of the most common zoonoses worldwide, and four of the six endemic species of brucellosis in animals can cause infections in humans [4]. The main route of infection in humans is consumption of contaminated milk and/or dairy products [5]. Patients with brucellosis exhibit a variety of nonspecific symptoms, such as fatigue, fever, and pain [6]. Complications of brucellosis can affect any organ system of the human body, causing disability and even death [7]. Human brucellosis treatment creates a heavy economic burden. Scholars have estimated that the cost of treatment for brucellosis is approximately $1,000 per patient [8].

China is one of the countries in which brucellosis is endemic. One study [9] was conducted with 5,483,076sheep in China from 2005 to 2016, and the results revealed 24,139 positive cases; the average positive rate was 0.44%. Lai’s analysis [10] showed that approximately 513,000 patients in mainland China had brucellosis from 1955 to 2014, and 90% of the cases occurred in the northern provinces of China; the incidence of human brucellosis was about 4.2 per 100,000 in 2014. Kong [11] described the prevalence and trends of human brucellosis in 15 provinces in southern China from 2004 to 2015, and the results showed that the incidence of human brucellosis in Guizhou, Yunnan, and Guangdong Provinces increased between 2012 and 2015.

Vaccine immunization, mandatory detection, culling, comprehensive monitoring and population health education are the primary measures for preventing and controlling animal brucellosis [12]. Although in certain countries, such as Canada and Japan, brucellosis eradication has met the World Organization for Animal Health (OIE) criteria, the disease is still a serious public health problem worldwide, especially when there are a large number of susceptible animals in a given region. The prevention and control of brucellosis is a public health problem [13]. Scholars [14] have noted that developing countries and regions, such as major epidemic areas, should focus on interdisciplinary and cross-domain cooperation in the prevention and control of brucellosis.

In 2016, the Ministry of Agriculture and National Health Commission of China jointly issued the “National Brucellosis Prevention and Control Plan (2016–2020),” proposing specific technical measures for the prevention and control of brucellosis and demanding that the principle of “government leads, departments collaborate, and all society participates” should be mandatory in the prevention and control of brucellosis [15].

From an economic perspective, investment will affect the effectiveness of the prevention and control of brucellosis. Studies [16, 17] have shown that if the investment of prevention and control is reduced after controlling brucellosis, the disease will re-emerge. Therefore, it is very important to establish a long-term investment mechanism and maximize the effectiveness of prevention and control.

In China, brucellosis prevention and control mainly depend on government investment. As livestock husbandry expands to production scale, consideration should be given to the supplementary role of private investment to achieve brucellosis prevention and control goals. Current studies [18–22] on influencing factors related to brucellosis mainly focus on the following: the prevalence of animal brucellosis; human brucellosis, especially in high-risk occupational populations; and curative effects on patients with brucellosis. However, very little research has been conducted on the state or influencing factors of private investment in brucellosis prevention and control. Our study aims to report the status of private investment in brucellosis prevention and control in epidemic areas of China and to explore the risk factors that may affect private investment.

## Materials and methods

### Study area

In mainland China, the provinces are divided into three classes by the Ministry of Agriculture according to the prevalence of brucellosis among animals and among humans:

First-class provinces: The incidence of human brucellosis is ≥1/10,000 or the number of epidemic counties of animal brucellosis/the total number of counties in the province is ≥30%.

Second-class provinces: The incidence of human brucellosis is <1/10,000 or the number of epidemic counties of animal brucellosis/the total number of counties in the province is <30%.

Third-class provinces: No cases of brucellosis are detected in humans or animals.

A cross-sectional, house-based study was conducted from April to October 2017 in 5 first-class brucellosis epidemic provinces—Inner Mongolia, Xinjiang, Shaanxi, Shanxi, Henan—and 2 second-class provinces—Guizhou and Guangxi—in China.

### Participants and sampling process

**Fig 1:**
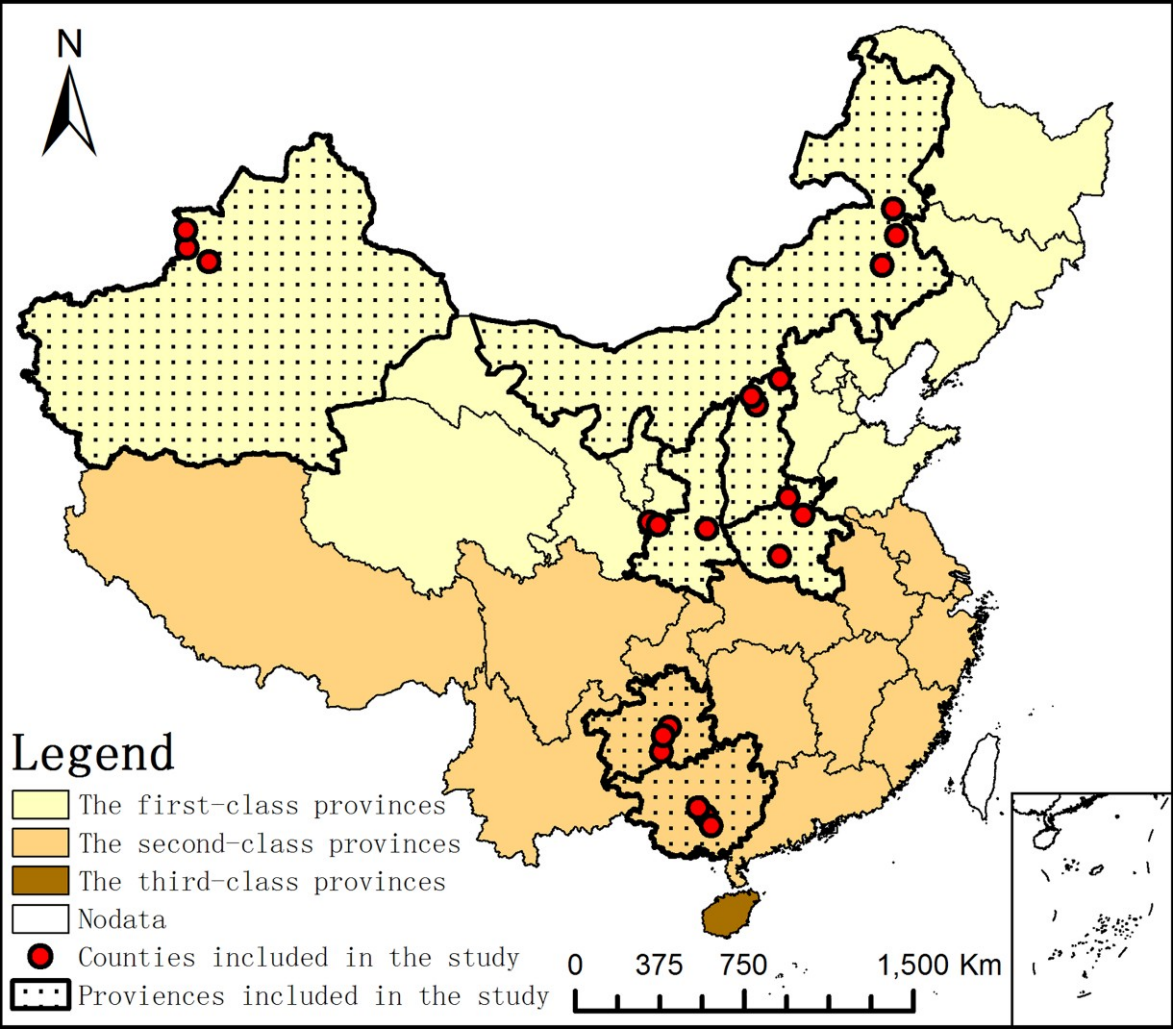
Map of the study area. Vector map file came from Resources and Environment Science Data Center, Chinese Academy of Sciences and was performed with ArcGIS10.4 software (ESRI INC., Redlands, CA, USA).

The inclusion and exclusion criteria were as follows: 1. The householder’s age is ≥ 18 years old. 2. Farming is the main business of the family, and sheep are the main species of livestock. 3. Families whose main business is to slaughter, sell or transport livestock are excluded. The full process of sample selection was as follows: Two counties with a higher prevalence of animal brucellosis were selected in each province. Then, 3 townships were randomly selected by a computer in each county, 5 villages were randomly selected in each township, and 5 sheep households were randomly selected in each village. As a result, 1050 households were screened in the study. (**Fig 1**).

### Ethics approval

The questionnaire survey received ethics approval from the Division of Epidemiology Survey within China Animal Health and Epidemiology Center (CAHEC) [23]. All participants were adult and signed a written informed consent after they fully understood the purpose and procedures of the study. There were no animal samplings in our study.

### Data collection

In this study, private investment refers to the monetary value of all production factors invested by sheep farmers in the process of brucellosis prevention and control.

A questionnaire survey was used to explore the potential influencing factors for farmers’ private investment in the prevention and control of brucellosis. The questionnaire was divided into five sections and comprised a series of questions pertaining to individual characteristics, farming experience, sheep farming income and investment of brucellosis prevention and control, brucellosis knowledge and behaviors and characteristics of behavioral economics. The questionnaire design and training of investigators were conducted by the CAHEC. The questionnaire is shown in S1.

### Statistical analysis

For assessing brucellosis knowledge, householders included were asked 25 questions. If the answer was yes, a score of 4 was assigned. The maximum accumulated score was 100. Four levels—<60, 60–69, 70–84, and 85–100—were used to indicate the degree of mastery of the respondents.

Means with standard deviation were calculated for continuous variables. Proportions were used for nominal variables. We used Chi-square test to compare rates and ANOVA for comparison of average private investment per sheep in provinces of different classes.

Private investment in each sheep was calculated as households’ annual investment in the prevention and control of brucellosis divided by the number of sheep kept. According to the “Compilation of Costs and Benefits of Agricultural Products in China,” $0.78 per sheep was taken as the reference level. In the logistic regression, an average investment per sheep of ≥ $0.78 was set to 1, whereas an investment of < $0.78 was set to 0. The average investment per sheep was changed to a dichotomous indicator, which was used as the dependent variable. Univariable logistic regression was carried out to select variables with *p* values<0.20, which were included in further multivariable models.

Statistical analysis was performed using SPSS Statistics Version 20.0 (IBM Corporation, USA). A 2-sided *p* < 0.05 was considered statistically significant.

## Results

### General findings

The response rate to the questionnaire was approximately 98.8% (1037/1050). An average age of the householders included in the study was 49.1±9.8 years. A total of 953 (91.9%) of them were males and the education level was mainly junior high school, with 556 persons (53.6%). The average number of family members was 4.3±1.6. (Table 1).

**Table 1:** Characteristics of households included in the study.

Type	Total	Average private investment per sheep ($)	
		≥0.78 (n = 287)	<0.78 (n = 750)
**Individual characteristics**			
Gender (male, %)	953(91.9)	256(89.2)	697(92.9)
Age (years), Mean (SD)	49.1(9.8)	50.3(7.1)	49.0(9.7)
Education level n (%)			
Primary school and below	280(27.1)	67(23.3)	213(28.4)
Junior high school	556(53.6)	155(54.0)	401(53.5)
High school	170(16.4)	53(18.5)	117(15.6)
College/undergraduate and above	31(3.0)	12(4.2)	19(2.5)
Family size, Mean (SD)	4.3(1.6)	4.4(1.6)	4.2(1.5)
**Sheep farming status**			
Herd size, Mean (SD)	128.9(175.4)	137.2(264.2)	107.5(183.1)
Sheep farming experience (years), Mean (SD)	9.8(8.8)	8.9(8.7)	9.9(8.8)
Grazing system n (%)			
Stocking	426(41.1)	96(33.4)	330(44.0)
Seasonal stocking	292(28.2)	93(32.4)	199(26.5)
Captive	319(30.8)	98(34.2)	221(29.5)
The percentage of sheep farming income of total household income (%), Mean (SD)	43.2(23.5)	71.4(13.9)	43.1(23.8)
**Brucellosis knowledge and behaviors**			
Score of related knowledge, Mean (SD)	70.4(22.7)	76.4(19.1)	61.3(23.7)
Actively learned related knowledge n (%)	516(50.2)	203(70.7)	313(41.7)
Paid close attention to related promotional campaigns n (%)	776(74.8)	240(83.6)	536(71.5)
Actively participated in related training courses n (%)	693(66.8)	247(86.1)	446(59.5)
**Characteristics of behavioral economics**			
Care about other people’s attitudes n (%)	372(35.9)	174(60.6)	198(26.4)
Exhibited donation behavior n (%)	916(88.3)	234(81.5)	682(90.9)
Satisfied with the policies n (%)	827(79.7)	254(88.5)	573(76.4)
Concerned about the health of neighbors’ livestock n (%)	814(79.4)	239(83.3)	575(76.7)
Supported the culling policy n (%)	649(72.6)	227(79.1)	422(56.2)

The average number of sheep raised by the households was 128.9±175.4 (10.0–1500.0). Among the surveyed households, 619 (59.7%) had a herd size ≤100. The years of sheep farming experience was 9.8±8.8. Furthermore, 426 (41.1%) households used a stocking grazing system. The percentage of sheep farming income out of total household income was 43.2 ± 23.5%. (Table 1).

The score of the householders was 70.4±22.7, and the proportion of positive answers of three questions of brucellosis prevention and control behaviors (S1 Part 4) was more than 50%. Approximately 64.1% of the heads of households surveyed did not care about other people’s attitudes in their daily life, while 79.4% of them were concerned about the health of neighbors’ livestock. Approximately 88.3% of the respondents conducted donation behavior. More than 70% of them were satisfied with the government’s animal disease prevention and control policy and supported culling (Table 1).

### Households’ private investment in the prevention and control of brucellosis

The average amount of private investment in the prevention and control of brucellosis was $0.73±0.54 per sheep, ranging from $0.14 to $2.23. There were 287 (27.7%) households with private investment at ≥ $0.78 per sheep (Table 1). The average amount of investment by farmers in the first-class provinces and the second-class provinces were $0.72 and $0.74, respectively (*p* = 0.221), and the reach rates were 27.63% and 27.46% (*p* = 0.093) (Table 2).

**Table 2:** The status of households’ private investment in the prevention and control of brucellosis in provinces of different classes.

Province	No. of households	No. of households reaching the reference level	Reach rate^1^ (%)	*p*^2^	Average private investment per sheep ($)	*p*^3^
First-class provinces	742	205	27.63	0.093	0.72	0.221
Inner Mongolia	148	44	28.83		0.68	
Shanxi	150	40	26.99		0.71	
Xinjiang	147	42	27.91		0.74	
Henan	150	41	29.15		0.70	
Guizhou	147	38	26.12		0.79	
Second-class provinces	295	81	27.46		0.74	
Guangxi	145	36	25.59		0.73	
Shaanxi	150	45	29.01		0.78	

1. Reach Rate = column 3/column 2*100

2. Chi-square test results for the reach rate of first-class provinces and second-class provinces

3. ANOVA results for the investment amount of first-class provinces and second-class provinces

### Analysis of factors associated with the amount of private investment on brucellosis prevention and control

In the univariable analysis, 17 variables were associated with the amount of private investment with a *p*-value < 0.2 and were selected for inclusion in the multivariate logistic regression analysis (Table 3). The facilitating factors included older age of householders, herd size >100, higher percentage of income from sheep farming of the total household income, higher score of brucellosis knowledge, actively learning related knowledge, actively participating in related training courses, care about others’ attitudes, concern about the health of neighbors’ livestock; the discouraging factor was support for the culling policy (Table 3).

**Table 3:** Factors associated with households’ private investment in the prevention and control of sheep brucellosis (univariable and multivariable analysis).

Variables	Univariate analysis		Multivariate analysis	
	OR (95%CI)	*p*	OR (95%CI)	*p*
Gender(male/female)	1.59(1.00–2.54)	0.050	1.93(0.67–5.52)	0.222
Age (Each additional one year old)	1.06(1.05–1.08)	0.000	1.07(1.03–1.11)	**0.001**
Education level				
Primary school and below	1.00		1.00	
Junior high school	1.23(0.88–1.71)	0.223	2.56(0.41–16.00)	0.314
High school	1.44(0.94–2.20)	0.093	2.42(0.41–14.25)	0.327
College/undergraduate and above	2.01(0.93–4.35)	0.077	2.30(0.36–14.87)	0.382
Number of family members				
≤4	1.00		1.00	
>4	1.12(1.03–1.22)		0.99(0.82–1.19)	0.914
Herd size				
≤100	1.00		1.00	
>100	3.33(2.51–4.42)	0.000	2.49(1.38–4.51)	**0.003**
Sheep farming experience(each additional year)	0.98(0.97–1.00)	0.072	1.00(0.97–1.04)	0.898
Grazing system				
Stocking	1.00		1.00	
Seasonal stocking	1.61(1.15–2.25)	0.006	0.63(0.30–1.31)	0.215
Captive	1.52(1.09–2.12)	0.012	0.63(0.30–1.29)	0.205
The percentage of raising sheep income of the total household income (Each additional 5%)	1.15(1.13–1.17)	0.000	1.14(1.11–1.16)	**0.000**
Score of related knowledge				
<60	1.00		1.00	
60–69	0.82(0.56–1.21)	0.325	1.38(0.62–3.09)	0.431
70–84	2.66(1.65–4.31)	0.000	3.49(1.48–8.16)	**0.009**
85–100	3.15(2.16–4.60)	0.000	3.85(1.40–10.51)	**0.004**
Actively learning related knowledge (Yes/No)	3.37(2.52–4.52)	0.000	2.98(1.55–5.74)	**0.001**
Close attention is paid to related promotional campaigns (Yes/No)	2.04(1.44–2.89)	0.000	1.90(0.40–3.01)	0.788
Actively participating in related training courses (Yes/No)	4.21(2.92–6.06)	0.000	3.07(1.52–6.18)	**0.002**
Care about the attitudes others (Yes/No)	2.12(1.86–2.42)	0.000	1.75(1.35–2.28)	**0.000**
Exhibited donation behavior (Yes/No)	0.56(0.45–0.69)	0.000	0.87(0.56–1.38)	0.561
Satisfied the policy (Yes/No)	0.63(0.53–0.76)	0.000	1.10(0.73–1.65)	0.662
Supported the culling policy (Yes/No)	0.56(0.49–0.65)	0.000	0.67(0.49–0.91)	**0.011**
Concerned about the health of neighbors’ livestock (Yes/No)	1.68(1.41–1.99)	0.000	1.75(1.23–2.51)	**0.000**

## Discussion

We conducted this cross-sectional study in seven provinces in China to survey the status of farmers’ private investment in the prevention and control of sheep brucellosis and analyze its influencing factors. Based on an investment of $0.78 per sheep, less than one-third of householders included in this study reached this level.

A previous study [24] indicated that brucellosis transmission from livestock to humans can be controlled through the sterilization of dairy and meat products, monitoring of brucellosis and improvements in feeding conditions, but large amounts of funding were required. Especially after a disease is controlled, reasonable private investment may need to be maintained and consolidated. For example, after Canada announced the elimination of brucellosis, one of the major problems was insufficient funding for future plans for the prevention and control of brucellosis.

From the perspective of economic theory, investment in the prevention and control of brucellosis in China presently has the characteristics of a public good; that is, the government plays a role as a manager in the input process, and all members share the benefits. However, this type of public good has drawbacks, such as regional imbalances and low resource utilization, which are limited by the amount of resources, regional development level and other conditions [25]. “The Lighthouse in Economics” demonstrates the potential for the private provision of public goods [26]. Private investment in the prevention and control of brucellosis can help supplement public investment. One study [27] from Iraq showed that when farmers invested $0.85 in brucellosis control in their flocks, the odds of *Brucella* seropositivity decreased by 10%.

The head of household makes and implements investment decisions for livestock breeding [28]; thus, individual characteristics such as gender, age, level of education, and number of family members were included in the regression analysis as possible influencing factors. The results indicated that old farmers invest more for brucellosis control because they are more aware of the hazards of brucellosis due to accumulated experiences of losses from the disease.

The regression analysis showed that the impact of herd scale on private investment in the prevention and control of brucellosis was statistically significant. Herd size is an important factor influencing the seroprevalence of brucellosis [29]. When the herd size is large, the increase in the risk of spreading disease stimulates farmers’ input in the prevention and control of brucellosis. In addition, compared with small-scale farmers, the coverage and completion of epidemic prevention measures was higher among large-scale farmers [30], which corresponded to greater investment in the prevention and control of animal epidemics.

The percentage of sheep farming income to total household income is an influencing factor. Sheep farming income is an important source of economic income for the farmers surveyed. Therefore, it can be inferred that when the proportion is higher, more attention is paid to the prevention and control of brucellosis, and the corresponding investment may increase.

Several related studies [31–34] have shown that susceptible populations, especially occupational contact populations, have a positive effect on the prevention of human brucellosis when they have adequate relevant knowledge. Additionally, the seroprevalence of brucellosis in the livestock raised may be lower when the relevant knowledge of the farmer is greater [35]. In our study, active participation in training courses and better mastery of knowledge may promote private investment by farmers.

Scholars have suggested [36] that farmers’ characteristics, especially socioeconomic characteristics, should be taken into consideration as an important part of future brucellosis prevention and control. In this study, based on the theory of behavioral economics [37], the behavioral and psychological characteristics of farmers were considered to affect private investment.

Householders who care about other people’s attitudes and are concerned about the health of neighbors’ livestock in their daily life have an increased probability of engaging in private investment. These two factors reflect the social psychology of farmers. When farmers pay more attention to the external environment, their investment in prevention and control may increase. This finding prompted us to create a positive prevention and control environment that may stimulate private investment.

Culling is one of the main strategies for controlling brucellosis [38]. An interesting observation is that a support on the culling policy is associated with lower probability of private investment reaching the reference level. This may be due to the fact that the more farmers invest, the greater the economic loss of sheep culling. Thus, to resolve the contradiction between supporting culling policy and private investment, a more reasonable culling policy must be needed.

### Strengths and limitations

This study was conducted in 5 first-class brucellosis epidemic provinces and 2 second-class brucellosis epidemic provinces in China. We surveyed farmers’ private investment in the prevention and control of brucellosis and added behavioral economics factors, together with individual characteristics and sheep farming characteristics, to regression analysis to explore the influencing factors. Sheep was the only livestock species used in this analysis to facilitate our investigation. In addition, the reference level of investment was obtained from statistical yearbook data. These limitations may affect the external authenticity of the study.

### Conclusion

Positive attitude towards brucellosis prevention and control, good knowledge of the disease, a larger sheep farming scale and a higher income from sheep farming are the main factors associated with higher private investment in brucellosis control. For intervention, providing knowledge of brucellosis is important for farmers' livelihoods.

## Supporting information

S1 Checklist Questionnaire. (PDF)

S2 Checklist STROBE checklist. (PDF)

## References

[1] Corbel M J. Brucellosis in humans and animals 1st ed Produced by the World Health Organization in collaboration with the Food and Agriculture Organization; 2006.

[2] Rubach MP, Halliday JEB, Cleaveland S, Crump JA. Brucellosis in low-income and middle-income countries. Curr Opin Infect Dis. 2013; 26(5):404–12. 10.1097/QCO.0b013e3283638104 23963260 PMC3888775

[3] Singh BB, Dhand NK, Gill JP. Economic losses occurring due to brucellosis in Indian livestock populations. Prev Vet Med [Journal Article]. 2015 2015-05-01; 119(3–4):211–5. 10.1016/j.prevetmed.2015.03.013 25835775

[4] Deng Y, Liu X, Duan K, Peng Q. Research progress on brucellosis. Curr Med Chem. 2018 2018-05-10. 10.2174/0929867325666180510125009 29745323

[5] Galinska EM, Zagorski J. Brucellosis in humans—etiology, diagnostics, clinical forms. Ann Agric Environ Med. 2013 2013-01-20; 20(2):233–8. 23772567

[6] Kose S, Serin SS, Akkoclu G, Kuzucu L, Ulu Y, Ersan G, et al Clinical manifestations, complications, and treatment of brucellosis: evaluation of 72 cases. Turk J Med Sci. 2014 2014-01-20; 44(2):220–3. 25536728 10.3906/sag-1112-34

[7] Buzgan T, Karahocagil MK, Irmak H, Baran AI, Karsen H, Evirgen O, et al Clinical manifestations and complications in 1028 cases of brucellosis: a retrospective evaluation and review of the literature. International Journal of Infectious Diseases. 2010; 14(6):e469–78. 10.1016/j.ijid.2009.06.031 19910232

[8] Rossetti CA, Arenas-Gamboa AM, Maurizio E. Caprine brucellosis: A historically neglected disease with significant impact on public health. PLoS Negl Trop Dis. 2017 2017-08-17; 11(8):e5692. 10.1371/journal.pntd.0005692 PMC5560528 28817647

[9] Cui Buyun, Jiang Hai. Surveillance data of brucellosis in China, 2005–2016. Disease Surveillance. 2018 2018-3-31; 33(3):188–192.

[10] Lai S, Zhou H, Xiong W, Gilbert M, Huang Z, Yu J, et al Changing Epidemiology of Human Brucellosis, China, 1955–2014. Emerg Infect Dis. 2017; 23(2):184–94. 10.3201/eid2302.151710 28098531 PMC5324817

[11] Kong W. Brucellosis infection increasing in Southern China. European Journal of Internal Medicine. 2018; 51:e16–8. 10.1016/j.ejim.2018.03.004 29549979

[12] Golshani M, Buozari S. A review of Brucellosis in Iran: Epidemiology, Risk Factors, Diagnosis, Control, and Prevention. Iran Biomed J. [Journal Article]. 2017 2017-11-01; 21(6):349–59. 10.18869/acadpub.ibj.21.6.349 28766326 PMC5572431

[13] Ducrotoy MJ, Bertu WJ, Ocholi RA, Gusi AM, Bryssinckx W, Welburn S, et al Brucellosis as an emerging threat in developing economies: lessons from Nigeria. PL oS Negl Trop Dis. [Journal Article; Research Support, Non-U.S. Gov't; Review]. 2014 2014-07-01; 8(7):e3008. 10.1371/journal.pntd.0003008 PMC4109902 25058178

[14] Franc KA, Krecek RC, Häsler BN, Arenas-Gamboa AM. Brucellosis remains a neglected disease in the developing world: a call for interdisciplinary action. BMC Public Health. 2018; 18(1). 10.1186/s12889-017-5016-y PMC5765637 29325516

[15] Ministry of Agriculture and Rural Affairs of the People’s Republic of China, National Health Commission of the People’s Republic of China. China Brucellosis Prevention Program (2016–2020).2016, Available from: http://www.moa.gov.cn/govpublic/SYJ/201609/t20160909_5270524.htm.

[16] Godfroid J. Brucellosis in livestock and wildlife: zoonotic diseases without pandemic potential in need of innovative one health approaches. Archives of Public Health. 2017; 75(1). 10.1186/s13690-017-0207-7 PMC5592711 28904791

[17] Caminiti A, Pelone F, Battisti S, Gamberale F, Colafrancesco R, Sala M, et al Tuberculosis, Brucellosis and Leucosis in Cattle: A Cost Description of Eradication Programmes in the Region of Lazio, Italy. Transbound Emerg Dis. [Journal Article]. 2017 2017-10-01; 64(5):1493–504. 10.1111/tbed.12540 27390169

[18] Marvi A, AsadiAliabadi M, Darabi M, et al Trend Analysis and Affecting Components of Human Brucellosis Incidence During 2006 to 2016[J]. Medical Archives, 2018, 72(1):17 10.5455/medarh.2018.72.17-21 29416212 PMC5789554

[19] Ayoola MC, Akinseye VO, Cadmus E, Awosanya E, Popoola OA, Akinyemi OO, et al Prevalence of bovine brucellosis in slaughtered cattle and barriers to better protection of abattoir workers in Ibadan, South-Western Nigeria. Pan Afr Med J. [Journal Article]. 2017 2017-01-20; 28:68 10.11604/pamj.2017.28.68.10925 29255538 PMC5726662

[20] Cash-Goldwasser S, Crump JA, Halliday JEB, Mmbaga BT, Maro VP, Kazwala RR, et al Risk Factors for Human Brucellosis in Northern Tanzania. The American Journal of Tropical Medicine and Hygiene. 2018 2018-02-07; 98(2):598–606. 10.4269/ajtmh.17-0125 29231152 PMC5929176

[21] Lytras T, Danis K, Dounias G. Incidence Patterns and Occupational Risk Factors of Human Brucellosis in Greece, 2004–2015. The International Journal of Occupational and Environmental Medicine. 2016 2016-10-01; 7(4):221–6. 10.15171/ijoem.2016.806 27651083 PMC6817955

[22] Kutlu M, Ergonul O, Sayin-Kutlu S, Guven T, Ustun C, Alp-Cavus S, et al Risk factors for occupational brucellosis among veterinary personnel in Turkey. Preventive Veterinary Medicine. 2014; 117(1):52–8. 10.1016/j.prevetmed.2014.07.010 25132061

[23] Zhou X, Li Y, Wang Y, Edwards J, Guo F, Clements AC, et al The role of live poultry movement and live bird market biosecurity in the epidemiology of influenza A (H7N9): A cross-sectional observational study in four eastern China provinces. J Infect. [Journal Article; Observational Study]. 2015 2015-10-01; 71(4):470–9. 10.1016/j.jinf.2015.06.012 26149187

[24] Roy S, McElwain TF, Wan Y. A network control theory approach to modeling and optimal control of zoonoses: case study of brucellosis transmission in sub-Saharan Africa. PLoS Negl Trop Dis. [Journal Article; Research Support, Non-U.S. Gov't; Research Support, U.S. Gov't, Non-P.H.S.]. 2011 2011-10-01; 5 (10):e1259 10.1371/journal.pntd.0001259 22022621 PMC3191122

[25] Jing SH, Fang Y, Wang F, et al Main Factors Influencing the Prevention and Control of Animal Epidemics and Countermeasures [J]. Hubei Journal of Animal Veterinary Sciences. 2017 2017-05-05(05):47–8.

[26] Luo SQ. On the Supply of Public Commodity—An Assessment on Coase’s “The Lighthouse in Economics” [J]. Huazhong Normal University Journal of Postgraduates. 2006 2006-06-15(02):125–7.

[27] Marvi A, AsadiAliabadi M, Darabi M, Abedi G, Siamian H, RostamiMaskopaee F. Trend Analysis and Affecting Components of Human Brucellosis Incidence During 2006 to 2016. Medical Archives. 2018; 72(1):17 10.5455/medarh.2018.72.17-21 29416212 PMC5789554

[28] Hassan OA, Affognon H, Rocklöv J, Mburu P, Sang R, Ahlm C, et al The One Health approach to identify knowledge, attitudes and practices that affect community involvement in the control of Rift Valley fever outbreaks. PLoS Negl Trop Dis. 2017 2017-02-16; 11(2):e5383. 10.1371/journal.pntd.0005383 PMC5332088 28207905

[29] Sagamiko FD, Muma JB, Karimuribo ED, Mwanza AM, Sindato C, Hang Ombe BM. Sero-prevalence of Bovine Brucellosis and associated risk factors in mbeya region, Southern highlands of Tanzania. Acta Tropica. 2018; 178:169–75. 10.1016/j.actatropica.2017.11.022 29191516

[30] Liu MY, Lu Q, Zhang SX. Farmers’ epidemic prevention and control behavior and its influencing factors with different feeding ways: Based on the survey data of 363 free-range farmers and scale breeding farmers [J]. Journal of Hunan Agricultural University (Social Sciences). 2016 2016-04-28(02):22–8.

[31] John K, Fitzpatrick J, French N, Kazwala R, Kambarage D, Mfinanga GS, et al Quantifying risk factors for human brucellosis in rural northern Tanzania. PLoS One. [Journal Article; Research Support, Non-U.S. Gov't]. 2010 2010-04-01; 5(4):e9968 10.1371/journal.pone.0009968 20376363 PMC2848606

[32] Sofian M, Aghakhani A, Velayati AA, Banifazl M, Eslamifar A, Ramezani A. Risk factors for human brucellosis in Iran: a case–control study. Preventive Veterinary Medicine. 2008; 12(2):157–61. 10.1016/j.ijid.2007.04.019 17698385

[33] Holt HR, Eltholth MM, Hegazy YM, El-Tras WF, Tayel AA, Guitian J. Brucella spp. infection in large ruminants in an endemic area of Egypt: cross-sectional study investigating seroprevalence, risk factors and livestock owner's knowledge, attitudes and practices (KAPs). BMC public health. 2011; 11(1):341. 21595871 10.1186/1471-2458-11-341 PMC3121632

[34] Awah-Ndukum J, Mouiche MMM, Bayang HN, Ngwa VN, Assana E, Feussom KJM, et al Seroprevalence and Associated Risk Factors of Brucellosis among Indigenous Cattle in the Adamawa and North Regions of Cameroon. Veterinary Medicine International. 2018; 2018:1–10. 10.1155/2018/3468596 PMC5817279 29535853

[35] Neilson WS. Theoretical Advances Spurred by “Stubborn Facts”. American Behavioral Scientist. 2011; 55(8):976–86.

[36] Kothalawala KAC, Makita K, Kothalawala H, Jiffry AM, Kubota S, Kono H. Association of farmers’ socio-economics with bovine brucellosis epidemiology in the dry zone of Sri Lanka. Preventive Veterinary Medicine. 2017; 147:117–23. 10.1016/j.prevetmed.2017.08.014 29254709

[37] Neilson WS. Theoretical Advances Spurred by “Stubborn Facts”. American Behavioral Scientist. 2011; 55(8):976–86.

[38] Moreno E. Retrospective and prospective perspectives on zoonotic brucellosis. Front Microbiol. 2014 2014-05-13; 5. 10.3389/fmicb.2014.00213 PMC4026726 24860561

