# Supplementary material for: Status and influencing factors of farmers’ private investment in the prevention and control of sheep brucellosis in China: A cross-sectional study
Source: PLoS Negl Trop Dis. 2019 Mar 25;13(3):e0007285. doi: 10.1371/journal.pntd.0007285 (PMC6448935; doi:10.1371/journal.pntd.0007285)
Supplement: S1 Checklist — (PDF) [file pntd.0007285.s001.pdf]

S1 Questionnaire Regarding Farmers' Private Investment In the Prevention and Control of Sheep Brucellosis

No.: Date: xxxx-xx-xx

Name of the Investigator: Work Unit: Phone Number:

Name of Householder: Phone Number:

Address: Province____ City ____County ____Town____ Village____

Part 1: Individual Characteristics

1. Gender: ① male, ② female

2. Age: ____ (years)

3. Number of family members:

4. Education Level: ① Primary School and Below; ② Junior High School; ③ High School; ④ College or Above.

Part 2: Farming Experience

1. You have raised sheep of about ____ years.

2. The number of sheep currently raised is ____.

3. Grazing system: ① Stocking (Barn Feeding)
   ② Seasonal Stocking (Seasonal Suitability Grazing)
   ③ Captive (Natural grazing)

Part 3: Sheep Farming Income and Investment of Brucellosis Prevention and Control

1. The proportion of sheep farming income to total household income every year is ____.

2. To prevent and control sheep brucellosis, the annual investment is about ______ (Unit: yuan) (Note: including the cost of purchasing services, consumables, etc.).

Part 4: Brucellosis Knowledge and Behaviors

Brucellosis knowledge

1. Is the sheep infected with brucellosis?

①Yes ② No ③ I do not know

2. What symptoms do you think appear after a sheep has been infected with

brucellosis?

(1) Abortion, premature delivery or placental retention

①Yes ② No ③ I do not know

(2) Ewe mastitis

①Yes ② No ③ I do not know

(3) Orchitic and epididymitis

①Yes ② No ③ I do not know

(4) Arthritis

①Yes ② No ③ I do not know

3. Is brucellosis in sheep transmitted to people?

①Yes ② No ③ I do not know

4. Can people infect each other?

①Yes ② No ③ I do not know

5. What clinical symptoms do you think people usually have after being infected with brucellosis?

(1) Long-term fever

①Yes ② No ③ I do not know

(2) Fatigue and sweating

①Yes ② No ③ I do not know

(3) Joint pain

①Yes ② No ③ I do not know

(4) Muscle pain

①Yes ② No ③ I do not know

6. Which of the following activities may cause infections in people?

(1) Direct contact with sick sheep

①Yes ② No ③ I do not know

(2) Drinking raw goat's milk and raw cheese

① Yes ② No ③ I do not know

(3) Eating uncooked sheep meat and offal

①Yes ② No ③ I do not know

(4) Inhalation of dust or aerosols containing contaminants

①Yes ② No ③ I do not know

7. Can brucellosis be prevented?

①Yes ② No ③ I do not know

8. Which of the following measures do you think will prevent the occurrence of sheep disease?

(1) Vaccine immunization

①Yes ② No ③ I do not know

(2) Newly purchased sheep need to pass quarantine and isolation

①Yes ② No ③ I do not know

(3) Preventing the flock from contacting or mixing with herds that are sick or of unknown status

①Yes ② No ③ I do not know

(4) Suspicious animals such as abortion and premature birth should be isolated until the diagnosis is clear.

①Yes ② No ③ I do not know

(5) Sheep placenta or stillbirth need to be burned or buried.

①Yes ② No ③ I do not know

9. Which of the following measures do you think can prevent the occurrence of human brucellosis?

(1) Regularly performing purification and disinfection work at the breeding site

①Yes ② No ③ I do not know

(2) Wear a full set of protective equipment for slaughtering operations

①Yes ② No ③ I do not know

(3) Do not consume unsterilized milk and dairy products

①Yes ② No ③ I do not know

(4) Do not eat raw or undercooked meat products

①Yes ② No ③ I do not know

Brucellosis prevention and control behaviors

1. Do you actively learn related knowledge?

①Yes ② No ③ I do not know

2. Do you always pay attention to the veterinary department's publicity activities?

①Yes ② No ③ I do not know

3. Do you actively participate in various training courses related to brucellosis prevention and control?

①Yes ② No ③ I do not know

Part 5: Characteristics of Behavioral Economics

1. Do you care about other people’s attitudes in life?

①Yes ② No ③ I do not know

2. Have you ever made a donation?

①Yes ② No ③ I do not know

3. Are you satisfied with the government's policies of brucellosis prevention and control?

①Yes ② No ③ I do not know

4. Do you support and cooperate with culling policy?

①Yes ② No ③ I do not know

5. Do you care about the health of neighbors’ livestock?

①Yes ② No ③ I do not know
