# Supplementary material for: Status and influencing factors of farmers’ private investment in the prevention and control of sheep brucellosis in China: A cross-sectional study
Source: PLoS Negl Trop Dis. 2019 Mar 25;13(3):e0007285. doi: 10.1371/journal.pntd.0007285 (PMC6448935; doi:10.1371/journal.pntd.0007285)
Supplement: S2 Checklist — (PDF) [file pntd.0007285.s002.pdf]

STROBE Statement—checklist of items that should be included in reports of observational studies

	Item No.	Recommendation	Page No.	Relevant text from manuscript
**Title and abstract**	1	(*a*) Indicate the study's design with a commonly used term in the title or the abstract	1	…a cross-sectional study
		(*b*) Provide in the abstract an informative and balanced summary of what was done and what was found	1,2	A total of 1037 households included in the study were analyzed.… Multivariable analysis showed that …
**Introduction**				
Background/rationale	2	Explain the scientific background and rationale for the investigation being reported	3,4,5	Brucellosis is a bacterial disease caused by… From an economic perspective, investment will impact the effectiveness of the prevention and control of brucellosis.…
Objectives	3	State specific objectives, including any prespecified hypotheses	6	Our study aims to …
**Methods**				
Study design	4	Present key elements of study design early in the paper	6,7,8,9	**Materials and methods…**
Setting	5	Describe the setting, locations, and relevant dates, including periods of recruitment, exposure, follow-up, and data collection	6,7,8,9	**Study area …** **Participants and sampling process…** **Data collection …** **Statistical analysis …** …
Participants	6	(*a*) *Cohort study*—Give the eligibility criteria, and the sources and methods of selection of participants. Describe methods of follow-up<br>*Case-control study*—Give the eligibility criteria, and the sources and methods of case ascertainment and control selection. Give the rationale for the choice of cases and controls<br>*Cross-sectional study*—Give the eligibility criteria, and the sources and methods of selection of	7	The inclusion and exclusion criteria were as follows: …

		participants		
		(*b*)*Cohort study*—For matched studies, give matching criteria and number of exposed and unexposed<br>*Case-control study*—For matched studies, give matching criteria and the number of controls per case		
Variables	7	Clearly define all outcomes, exposures, predictors, potential confounders, and effect modifiers. Give diagnostic criteria, if applicable	7,8,9	According to the "Compilation of Costs and Benefits of Agricultural Products in China," $0.78 per sheep was taken as the reference level…
Data sources/measurement	8*	For each variable of interest, give sources of data and details of methods of assessment (measurement). Describe comparability of assessment methods if there is more than one group	8	**Data collection …**
Bias	9	Describe any efforts to address potential sources of bias	7,8	The questionnaire was designed by CAHEC**…**
Study size	10	Explain how the study size was arrived at	7	The full process of sample selection was as follows…

Continued on next page

Quantitative variables	11	Explain how quantitative variables were handled in the analyses. If applicable, describe which groupings were chosen and why	8	**Statistical analysis …**
Statistical methods	12	(*a*) Describe all statistical methods, including those used to control for confounding	9	**Statistical analysis …**
		(*b*) Describe any methods used to examine subgroups and interactions	9	**Statistical analysis …**
		(*c*) Explain how missing data were addressed	9	The response rate to the questionnaire was…
		(*d*) *Cohort study*—If applicable, explain how loss to follow-up was addressed<br>*Case-control study*—If applicable, explain how matching of cases and controls was addressed<br>*Cross-sectional study*—If applicable, describe analytical methods taking account of sampling strategy	7	The full process of sample selection…
		(*e*) Describe any sensitivity analyses	15,16	Table 3
**Results**				
Participants	13*	(a) Report numbers of individuals at each stage of study—eg numbers potentially eligible, examined for eligibility, confirmed eligible, included in the study, completing follow-up, and analysed	10	…The response rate …98.8%...
		(b) Give reasons for non-participation at each stage	no	
		(c) Consider use of a flow diagram	no	
Descriptive data	14*	(a) Give characteristics of study participants (eg demographic, clinical, social) and information on exposures and potential confounders	12-16	Table 1,2,3
		(b) Indicate number of participants with missing data for each variable of interest	no	
		(c) *Cohort study*—Summarise follow-up time (eg, average and total amount)		
Outcome data	15*	*Cohort study*—Report numbers of outcome events or summary measures over time		
		Case-control study—Report numbers in each exposure category, or summary measures of exposure		
		Cross-sectional study—Report numbers of outcome events or summary measures	10	…was approximately 98.8% (1037/1050) .
Main results	16	(*a*) Give unadjusted estimates and, if applicable, confounder-adjusted estimates and their precision (eg, 95% confidence interval). Make clear which confounders were adjusted for and why they were included	15,16	**Table 3**.
		(*b*) Report category boundaries when continuous variables were categorized	15,16	**Table 3**
		(*c*) If relevant, consider translating estimates of relative risk into absolute risk for a meaningful time period	no	

Continued on next page

Other analyses	17	Report other analyses done—eg analyses of subgroups and interactions, and sensitivity analyses	no	
**Discussion**				
Key results	18	Summarise key results with reference to study objectives	17-19	…Based on an investment of $0.78 per sheep, less than one-third of householders included in this study reached this level<br>…<br>…The regression analysis showed that…
Limitations	19	Discuss limitations of the study, taking into account sources of potential bias or imprecision. Discuss both direction and magnitude of any potential bias	20	…These limitations may affect the external authenticity of the study.
Interpretation	20	Give a cautious overall interpretation of results considering objectives, limitations, multiplicity of analyses, results from similar studies, and other relevant evidence	20	Conclusion…
Generalisability	21	Discuss the generalisability (external validity) of the study results	20	…For intervention, providing knowledge of brucellosis is important for farmers' livelihoods …
**Other information**				
Funding	22	Give the source of funding and the role of the funders for the present study and, if applicable, for the original study on which the present article is based	no	

*Give information separately for cases and controls in case-control studies and, if applicable, for exposed and unexposed groups in cohort and cross-sectional studies.

Note: An Explanation and Elaboration article discusses each checklist item and gives methodological background and published examples of transparent reporting. The STROBE checklist is best used in conjunction with this article (freely available on the Web sites of PLoS Medicine at http://www.plosmedicine.org/, Annals of Internal Medicine at http://www.annals.org/, and Epidemiology at http://www.epidem.com/). Information on the STROBE Initiative is available at www.strobe-statement.org.
